# Pension Tension: Retirement Annuity Fosters Ageism Across Countries and Cultures

**DOI:** 10.1093/geroni/igad080

**Published:** 2023-07-27

**Authors:** Zizhuo Chen, Michael S North, Xin Zhang

**Affiliations:** School of Psychological and Cognitive Sciences, Beijing Key Laboratory of Behavior and Mental Health, Peking University, Beijing, China; Department of Management and Organizations, New York University, New York, New York, USA; School of Psychological and Cognitive Sciences, Beijing Key Laboratory of Behavior and Mental Health, Peking University, Beijing, China

**Keywords:** Ageism, Future-self intervention, Intergenerational tensions, Public pensions

## Abstract

**Background and Objectives:**

Globally aging populations raise worldwide concerns about how an older population will be valued. Cross-culturally, many espouse that Eastern cultures revere their older adults more than Westerners, due to stronger collectivism and filial piety traditions. In contrast, this paper proposes a resource tension hypothesis, whereby rapid population aging causes pragmatic strain across all modernized societies, fostering ageism.

**Research Design and Methods:**

Three studies supported this resource tension hypothesis, focusing on the pragmatic role of public pensions—a fundamental resource inherently pitting older versus younger generations—in fostering ageism. Study 1 tested the relationship between nation-level public pension rate and attitudes toward older adults by using World Values Survey and European Social Survey data sets. Study 2 further explored this relationship via priming both the pension-based resource scarcity and the intergenerational competition over the public pension. Study 3 offered an intervention—future-self-thinking via a photo ager—on reducing intergenerational tensions under pension scarcity conditions.

**Results:**

Study 1 found a significant link between nation-level public pension rate and negative older adult attitudes across 39,700 World Values Survey, and 29,797 European Social Survey data points. Study 2 further supported the pension-ageism link via experimental methods. Participants who were reminded of the scarcity of pensions and intergenerational competition exhibited more negative attitudes toward older adults. Study 3 confirmed the effect of the future-self intervention on enhancing attitudes toward older adults even despite scarce pension resources.

**Discussion and Implications:**

The findings support a resource explanation in driving perceptions of older adults, implicate pensions as a key mechanism driving intergenerational attitudes, and identify future-self thinking as a critical intervention. The present studies open up new research pathways for understanding and accommodating the globally aging population.

**Translational Significance:** In the present study, we propose and test a Resources Tension Hypothesis, through the lens of public pensions. The results indicated that public pension insufficiency could indeed provoke intergenerational tension (i.e., negative attitudes toward older adults), even in a collectivistic culture such as China. Nevertheless, a new future-self intervention was confirmed to offset the harmful effect of pensions on ageist attitudes, such that people with future-self imagination manipulation were no longer influenced by pension insufficiency in terms of their attitudes toward older adults. The effect of future-self continuity on reducing generational tension has timely implications in an aging, multigenerational world.

The world is aging rapidly. There exist 841 million over the age of 60, more than four times higher than in 1950, and this figure projects to reach two billion by 2050 (United Nations, 2013). These demographic aging trends present emergent, pragmatic concerns, including accommodating an aging workforce and conceptualizing attitudes toward older adults (North & Fiske, 2015a, 2015b). All these concerns draw attention to ageism (targeting older adults), which includes both the attitudinal and behavioral components. Specifically, ageism was first conceptualized by Butler (1969) and then defined as “a process of systematic stereotyping and discrimination against people because they are old” (Butler, 1975) and is presented nowadays in both our “perceptions of older people” and “actions towards older people” (Ayalon & Tesch-Römer, 2018, p. 1). Based on these prevalent perspectives stressing the stereotype against and perceptions of older adults (i.e., the attitudinal component), we operationalize *ageism* in the present study as people’s attitudes toward older adults according to the Stereotype Content Model (Fiske et al., 2002). Given that aging trends persist globally (Lutz et al., 2008), the association between demographic aging and ageist attitudes is a virtually universal concern.

## From Cultural-Value Explanation to Resource Tension Hypothesis

Given the impetus to understand global attitudes toward older adults, scholars have often pondered whether ageism might be more pronounced in certain cultures than others. In positing one answer, some scholars have endorsed a cultural-value explanation that Eastern cultures might exhibit less ageism than Western cultures. This view stems primarily from the idea that Eastern (versus Western) cultures are more interdependent, prioritizing relational harmony over individual success (Nisbett, 2004). Indeed, Easterners do hold social groups responsible for the behaviors and outcomes of individuals (Morling & Fiske, 1999) such as the greater tradition of *filial piety*—the obligation to respect, obey, and care for older adults that spans various Eastern traditions (Koyano, 1996; Ng, 1998, 2002; North & Fiske, 2012; Singh, 2005; Sŏng & Kim, 2009; Sung, 1995). Therefore, this perspective believes that—due to stronger historical norms of collectivism and filial piety—Eastern cultures (e.g., China) should express greater positive attitudes toward older adults than Western cultures (e.g., United States, Ackerman & Chopik, 2021; Levy & Langer, 1994; Nelson, 2009). By extension, older adults might be more readily accommodated in Eastern cultures, in contrast to the West’s historic emphasis on independence, which helped foster older adults’ largely role-less, retired state (North & Fiske, 2012).

However, recent studies have challenged this cultural-values assumption. For example, although ageism differed cross-culturally, it was masculinity and the long-term orientation, not collectivism, that predicted these differences (Ng & Lim-Soh, 2021). One meta-analysis found that Easterners instead held more negative attitudes toward older adults than Westerners, overall (mean effect size = −0.31), and that collectivism actually predicted more negative views, not positive ones (North & Fiske, 2015b). Cross-cultural evidence further claimed that priming personal values rather than cultural values could influence ageist attitudes more (Zhang et al., 2016).

In contemplation of the mixed evidence of the culture-ageism causality, we propose an alternative, *resource tension hypothesis* underlying the national differences of ageism. This theory posits that modern realities stemming from an aging population transcend cultural values in shaping current attitudes toward older adults (North & Fiske, 2015a)—that is, even though certain cultures may have featured historically greater expectations for respecting older adults, these expectations pale in comparison to the pragmatic resource strain stemming from an unprecedented aging population (North & Fiske, 2015b). Specifically, an aging population spanning both Eastern and Western societies raised concerns over the availability of resources between generations, regardless of cultural norms (North & Fiske, 2012). Scholars have cited rapid population aging as a primary harbinger of economic downturns (Longman, 2005). Similarly, others argue that population aging will result in resource scarcity saliency, fostering negative attitudes toward older adults (North & Fiske, 2013b).

Additionally, we test this hypothesis through the lens of public pension resources (known as “pay-as-you-go” pension), but not the private pension system (known as the fully funded pension), because the latter might not fit with the present study’s purpose. Because public pension resources come from taxes paid by the working population (usually the younger generations), although the latter depends mainly on personal savings from individual accounts. As a result, such public pension system pits older generations’ benefits (i.e., who reap these rewards upon imminent retirement) versus younger generations’ (i.e., who pay into these rewards with the expectation of eventually enjoying them upon retirement). Moreover, rapid, global population aging further threatens existing public pension systems’ sustainability (Bonoli, 2005), which might exacerbate such intergenerational tension.

Ultimately, this paper argues for a generational resource tension (over cultural values) perspective, through the lens of pensions, an urgent policy issue. The findings present timely considerations and new avenues for research within the increasingly aging, multigenerational workforce, spanning countries, and cultures worldwide.

### The Aging Population Raises Intergenerational Resource Tensions

Integrated threat theory (Croucher, 2017; Stephan & Stephan, 2000) suggests that the ingroup perceives realistic threats from the outgroup concerning scarce economic and political resources. Given the globally aging population, scholars have found that intergenerational competition for public resources could foster younger adults’ negative attitudes toward older adults, such as perceiving them as particular realistic threats (Ayalon, 2019). First of all, spanning industrialized countries around the world, rapid population aging has yielded a growing imbalance between the global working-age population (16–65) and the older population (65+; Society for Human Resource Management, 2015). This unevenness signified an increasing burden on younger generations to work in support of older ones (Mason et al., 2017) and was exemplified by a ratio of workers to pensioners expected to decrease by 50% over the next few decades, from 4-to-1 to 2-to-1 (Quattrucci, 2017). Pragmatically speaking, the increasing older population has been viewed as a burden to society (Hövermann & Messner, 2023), especially when resource scarcity is salient (Cohn-Schwartz & Ayalon, 2021; Francioli et al., 2023).

Moreover, public pension systems directly pit the benefits of older generations versus newer ones and form the “generational contract” that underlies societies around the globe (Engelen, 2003; Svallfors, 2008). But for the first time ever, demographic forces around the world are raising the real possibility that younger generations will never eventually reap this resource in later life (Bonoli, 2005; Serrano et al., 2011; Zaidi, 2010) because an aging population already risks general economic downturn (Bloom et al., 2010) and threatens the proverbial generational contract. Given that younger adults held expectations for older adults to step aside and make way for younger generations (North & Fiske, 2013a), it stands to reason that an emphasis on public pensions might particularly stoke intergenerational fires, because it is a resource directly at risk of dissipating with an aging population.

Nevertheless, despite these emergent concerns, a link between pension scarcity and ageism—and whether this link persists across cultures—has, to our knowledge, not been established. Given rapid population aging concerns around the world, a major question surrounds how older adults were valued across cultures. From this standpoint, we propose the *resource tension hypothesis,* in which the pragmatic resource strain presented by the aging population resulted in negative attitudes toward older adults across different countries with different cultural values.

### The Resource Tension Hypothesis: Dose Resource (Pension) Tension Lead to Ageism?

Previous studies provide both macro and micro-level evidence on how population aging might be linked with younger adults’ negative perceptions of older adults. *Macro-level* perspectives usually suggest a negative association between the degree of population aging and the general attitudes toward older adults at the national level. One large-scale analysis, including data from 26 nations comprising all six populated continents, found that population aging predicts negative attitudes toward older adults (Löckenhoff et al., 2009). By analyzing a large database of 400 million words, Ng et al. (2015) found that every 1% increase in the proportion of older adults over 65 years contributed to a 12.4% increase in negativity of age stereotypes per decade. As noted, one meta-analysis synthesizing 41 papers, 23 countries, and over 21,000 data points, similarly linked recent population aging rises and negative attitudes toward older adults—and that Easterners reported more negative attitudes toward older adults than did Westerners, overall (North & Fiske, 2015b). In the same meta-analysis, a resource-based ageism perspective was proposed to explain such (East vs West) cultural differences. A more recent study further confirmed that older adults are more frequently perceived as a social burden in faster-aging countries (Hövermann & Messner, 2023). Thus, unlike the cultural values prediction—and unlike traditional “contact hypothesis” predictions that greater exposure to outgroups generated greater positivity toward them (Dovidio & Gaertner, 1999)—a resource tension hypothesis predicts that the increased presence of older adults fosters negative attitudes toward them.

Nevertheless, these analyses suggest only indirectly that resource scarcity concerns (using population aging as a proxy thereof) underlie ageism. To more directly implicate resource tensions in fostering ageism, the current paper tested whether public pension resources—which directly pit older and younger generations against one another—predict ageism at the national level. Specifically, in order to account for macro-level variables such as cultural values or socioeconomic development status, we introduce both Western and Eastern countries with differences in a variety of macro-level variables (such as GDP [gross domestic product] per capita, Gini coefficient, and individualism scores) in the same analysis when testing this resource tension hypothesis.

*Hypothesis 1 (H1): A macro-level link between public pension resources and attitudes towards older adults exists across countries.*

This hypothesis also warrants *micro-level* exploration that reminding people of scarce pension resources will alternate individual attitudes towards older adults. To our knowledge, perceived generational resource scarcity and ageism were first directly connected by North and Fiske (2012), centered in the United States. Broadly, this argument posited that younger generations resent older generations who appear to use up resources at the expense of younger ones (North & Fiske, 2013c), especially under conditions of general resource scarcity (North & Fiske, 2016). During the COVID-19 outbreak, intergenerational competition on limited public health resources aggravated intergenerational tension and resulted in worsening ageism against older adults (Ayalon, 2020; Lytle & Apriceno, 2022; Sutter et al., 2022). The current paper extended this line of thinking, exploring whether manipulation of perceived *pension* scarcity would further exacerbate ageism toward older adults, and within a traditionally collectivistic culture. As discussed earlier in this paper, pensions provide a unique testing ground, in that they are a worldwide resource directly placing the interests of older versus younger generations.

*Hypothesis 2 (H2): A micro-level link between pension resource scarcity and attitudes towards older adults will emerge even within a collectivistic culture.*

### Mitigating Generational Resource Tensions Via a Future-Self Intervention

Additionally, we identify a critical missing piece within this literature: an ageism intervention. Although scholars have encouraged reliable ageism interventions such as positive education about aging or contact experiences (i.e., PEACE, Lytle & Levy, 2019; Lytle et al., 2021), younger adults do not always have opportunities to join long-term education programs or maintain close contact with older adults.

The present paper also tests a novel and more feasible intervention toward enhancing attitudes toward older adults: Future-self thinking. By inviting younger adults to imagine what they would feel, think, do, or be like in their 70s, younger adults might come to enact more positive attitudes toward older adults (Packer & Chasteen, 2006). Prior work shows the surprising potency of encountering one’s future-self in enhancing later-life concerns, particularly retirement savings (Hershfield et al., 2011). However, this type of intervention fails to alleviate ageism if younger adults stuck to their identification (as the young, Packer & Chasteen, 2006) or if they looked at the digital-processed photos of the aging self (Rittenour & Cohen, 2016). One possibility for such null effects may be that younger adults’ general attitudes toward older adults are not negative enough to be significantly influenced in the first place. In other words, younger adults would only express their negative attitudes toward older adults under certain (e.g., resource scarcity) circumstances, in which interventions are more relevant. Thus, by adapting this paradigm to the current paper’s focus, we explore how a future-self mentality might provide a salve for beliefs surrounding older and younger scarce pension resource competition.

*Hypothesis 3 (H3): The pension scarcity-ageism link will be attenuated by facing one’s future self.*

## Study 1: Pension Rate and Attitudes Toward Older Adults

Using two different cross-national data sets, we tested the prediction that nation-level public pension resources foster ageism at the national level (Hypothesis 1). We explored whether the national public pension rate would correlate with national attitudes toward older adults—and moreover, mediate the relationship between the proportion of the older population and attitudes toward older adults in each country.

### Method

#### Data source

We obtained the World Values Survey data set (WVS, available at www.worldvaluessurvey.org), the sixth wave, conducted between the years 2010 and 2014, and European Social Survey (ESS, available at https://ec.europa.eu/eurostat) conducted in 2008, fourth wave. We chose these waves because they were the only ones to include attitudes toward older adults. The WVS data included 39,700 individuals from 29 nations worldwide (including both European and non-European countries, 51% female, *M*_age_ = 43.16, *SD*_age_ = 16.94). The ESS data included 29,797 individuals from 20 EU countries (53.3% female, *M*_age_ = 48.37 years, *SD*_age_ = 17.91 years). In ESS data sets, non-EU country data, comprising 22.26% of the whole survey, were excluded due to a lack of reliable pension-related data in the year 2008, when the fourth wave of ESS was conducted. Detailed descriptive statistics appear in Supplementary Tables 1 and 2.

### Material and Measurements

#### Attitudes toward older adults

Ageism comprises perceptions of older people (i.e., aging stereotype) and actions toward them (Ayalon & Tesch-Römer, 2018, p. 1). Nevertheless, Study 1 used the former element only as the indicator of ageism, because younger adults’ attitudes towards older adults according to the Stereotype Content Model was the only available ageism metric. In both WVS and ESS data sets, participants rated the likelihood of most people in their country viewing those over 70 as *friendly* (i.e., the warmth dimension in the Stereotype Content Model) or *competent* (i.e., the competence dimension) from 0 (*“Not at all likely”*) to 4 (*“very likely”*). For parsimony, as well as avoiding biased estimation with single-item measure, analyses collapsed the attitudes into one index, averaging the two scores on warmth and competence items (α = 0.59 for WVS and α = 0.58 for ESS).

#### Demographic information

Participants reported their *gender* (1 = “male”; 2 = “female”), *age*, and *education level* (from 1 = “no formal education” to 9 = “university-level education” in WVS, and from “1 = less than lower secondary education (ISCED 0–1)” to “5 = tertiary education (ISCED 5-6)” in ESS), as well as *self-reported income* on a 10-point scale (from 1 = “lowest” to 10 = “highest” in WVS, and from 1 = “1st decile” to 10 = “10th decile” in ESS).

#### Percentage of public pension expenditure in GDP

For each country in the present study, we calculated the percentage of pension expenditure in GDP in 2008 or 2010, depending on whether the country presented in the WVS (conducted from 2010 to 2014) or ESS (conducted in 2008) data set. The national public pension expenditure and GDP information were derived from the World Bank open data (https://data.worldbank.org/) and the Eurostat data set (https://www.pordata.pt/en/Europe).

#### Other country-level variables

We controlled each country’s score on the cultural-value dimension of *individualism* (0–100; Hofstede, 2001). Given the resource tension hypothesis tested in the present study, we also controlled the *GDP per capita* and *Gini coefficient* (a measure of income inequality ranging from 0 to 1, which higher values indicate higher inequality) as the indicator of an overall level of the resource and its allocation within each country. In addition, we collected the *proportion of older adults over 60 in the total population* as another country-level independent variable because the previous studies demonstrated the relationship between the proportion of older adults and ageist attitudes (Ng et al., 2015; North & Fiske, 2015b). In the present study, we suggested that the proportion of older adults was an antecedent of the pension expenditure, which could in turn harm the attitudes toward older adults. All these data were derived from the World Bank Open Data in either 2008 or 2010 to match our ESS or WVS data sets, respectively.

#### Analysis

We conducted hierarchical linear modeling (HLM) to examine the association between pension and attitudes toward older people with Mplus (Muthén & Muthén, 1998–2017). We standardized all individual-level and country-level variables before entering them into the model. We also tested the potential mediating effect of pension resources in explaining the association between the percentage of the older population and attitudes toward older adults.

### Results and Discussion

HLM results (Table 1) indicated that consistent with previous studies, the proportion of older adults in each country negatively correlated with attitudes toward older adults (WVS: *b* = −0.014, *t* = −23.149, *p* < .001; ESS: *b* = −0.053, *t* = 9.078, *p* < .001) in the corresponding country.

**Table 1.** Hierarchical Linear Modeling Analysis of the Association Between Attitudes Toward Older Adults and Individual- and Country-level Variables, and Mediation Effects Through Pension Expenditure (Study 1)

Variable	Based on WVS data					Based on ESS data				
	*b*	*SE*	Estimated effect	95% CI		*b*	*SE*	Estimated effect	95% CI	
				LL	UL				LL	UL
Individual level										
*Attitudes towards older adults* as dependent variable										
Age	0.003**	0.001		0.002	0.004	0.003**	0.001		0.002	0.005
Gender	−0.040**	0.011		−0.061	−0.019	−0.037*	0.012		−0.059	−0.014
Education level	−0.001	0.005		−0.014	0.009	−0.041**	0.006		−0.053	−0.030
Income	0.011*	0.004		0	0.019	−0.003	0.003		−0.008	0.002
Country level										
*Attitudes towards older adults* as dependent variable										
Intercept	1.066	1.059				−1.292	1.974			
Individualism score	−0.003	0.002		−0.008	0.002	Null	Null		Null	Null
GDP per capita	0.006	0.003		0.000	0.012	0.042	0.041		−0.038	0.123
Gini coefficient	−0.004	0.004		−0.012	0.003	−0.008	0.013		−0.033	0.017
Proportion of older adults	−0.016	0.010		−0.036	0.004	−0.015	0.018		−0.050	0.020
Pension *(a)*	−0.027*	0.013		−0.053	−0.002	−0.039*	0.013		−0.065	−0.014
*Pension expenditure* as dependent variable										
The proportion of older adults *(b)*	0.369**	0.042		0.288	0.451	0.404*	0.195		0.023	0.785
Indirect effect (a*b)			−0.010*	−0.020	0.000			−0.016*	−0.032	0.000

*Notes:* CI = confidence interval; ESS = European Social Survey; GDP = gross domestic product; HLM = hierarchical linear model; LL = lower limit; SE = standard error; UL = upper limit; WVS = world values survey. Individualism was not analyzed in the HLM model on ESS (2008), because we failed to find the matched data.

*p < .05. **p < .001.

**Figure 1.**
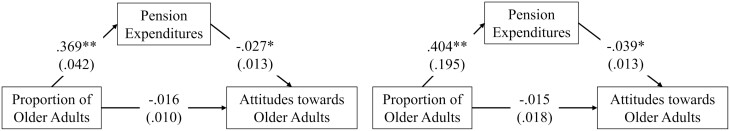
Mediation model of pension expenditure (%GDP) on the effect of proportion of older adults to attitudes towards older adults with (A) WVS data and (B) ESS data in Study 1. The standard errors of path coefficients were presented in parentheses. ESS = European Social Survey; GDP = gross domestic product; WVS = World Values Survey. *p < .05; *p < .001.

Subsequent mediation analysis revealed that country-level public pension expenditure was positively associated with the country-level percentage of older adults (WVS: *b* = 0.369, *t* = 8.862, *p* < .001; ESS: *b* = 0.404, *t* = 2.077, *p* = .038), and negatively associated with country-level attitudes toward older adults (WVS: *b* = −0.027, *t* = −2.119, *p* = .034; ESS: *b* = −0.039, *t* = −3.072, *p* = .002). After adding pension into the model, the association between the percentage of older adults and attitudes toward older adults became nonsignificant, (WVS: *b* = −0.016, *t* = −1.571, *p* = .116; ESS: *b* = −0.015, *t* = −0.846, *p* = .398), suggesting potential mediation effect of pension rate on the association between the percentage of older adults in the total population and attitudes toward older adults. This was further confirmed with a Monte Carlo simulation, mediation index *b* = −0.010, *t* = −1.997, *p* = .046, 95% CI = (−0.017, −0.003; WVS), *b* = −0.016, *t* = −1.998, *p* = .046, 95% CI = (−0.032, ≤0.001; ESS; Figure 1A and 1B).

Replicating previous findings (Löckenhoff et al., 2009; North & Fiske, 2015b; Zhang et al., 2016), Study 1 found that the older population proportion negatively correlated with attitudes toward older adults. However, the current study goes beyond this existing work by implicating the public pension rate as a mechanism explaining this relationship after both individual and cultural level values were controlled. These results provide initial support for the resource tension hypothesis (H1).

## Study 2: An Experimental Examination Between Pension Salience and Attitudes Toward Older Adults

In the present study, we reminded younger and middle-aged adults of both *pension system solvency* (sufficient vs insufficient) and *generational competition over these resources* (with intergenerational competition vs without intergenerational competition). Moreover, we implemented a within-subjects design to further reduce the influence of individual differences in attitudes toward older adults. Based on the resources tension hypothesis, we predicted that priming the salience of the pension crisis would exacerbate ageist attitudes toward older adults, even within an Eastern culture—and moreover, that salience of resource competition would further lower younger adults’ attitudes toward older adults (H2).

### Method

Before data collection, a power analysis was conducted for sample size determination using G*power software. This analysis recommended at least 64 participants on conducting repeated measurement ANOVA (we set the effect size equal to 0.25, α error probability equal to 0.05, and test power equal to 0.90). We recruited 275 Chinese younger and mid-aged adults (56% females, *M*_age_ = 33.39, *SD*_age_ = 7.26) from a local comprehensive university, as well as from local companies via convenience sampling in the year 2016. Participants received course credit or ￥50 (equal to $8) monetary reward for their participation in the present study.

The data comprised two waves. In the first wave, participants completed a four-item, older adult perception measure derived from previous research on the Stereotype Content Model (Caprariello et al., 2009). Participants used a 7-point Likert-like scale (from 1= “totally not appropriate” to 7 = “totally appropriate”) to rate the extent to which four adjectives describe a typical person over 65 years of age. Two of the adjectives (competent and capable) measured the *competence* dimension, although the other two (warmth and friendly) measured the *warmth* dimension; the measure was used in prior work, yielding adequate validity (Fiske et al., 2002; Zhang et al., 2016). We collapsed these four measures into one index representing overall attitude toward older adults (for pre-test, α = 0.77; for post-test, α = 0.84; control variables, none of which showed significance, appear in Supplemental Materials).

Approximately two weeks later, participants completed the second wave. All participants read one of four short articles ostensibly taken from a local newspaper, generated specifically for this study. Articles reported on China’s pension system (i.e., the national public pension, same as the pension concept used in Study 1), stating that, according to a recently released government report, the system either is facing a huge financial gap (*insufficient* condition) or does not face any financial challenge for the foreseeable future (*sufficient* condition). Next, the article stated clearly either that the pension would be funded by the younger population’s tax (*with intergenerational competition* condition) or did not specifically mention the source of the pension (*without intergenerational competition* condition). Participants were randomly assigned to one of these four conditions (2 levels of sufficiency × 2 levels of the salience of competition) and given 2 min to read the story and generate a proper title for it (as a manipulation). After reading the story, participants completed filler questionnaires, followed by the same measure of attitudes toward older adults as the first wave (controlling variables collected could be found in Supplemental Materials).

### Results and Discussion

**Figure 2.**
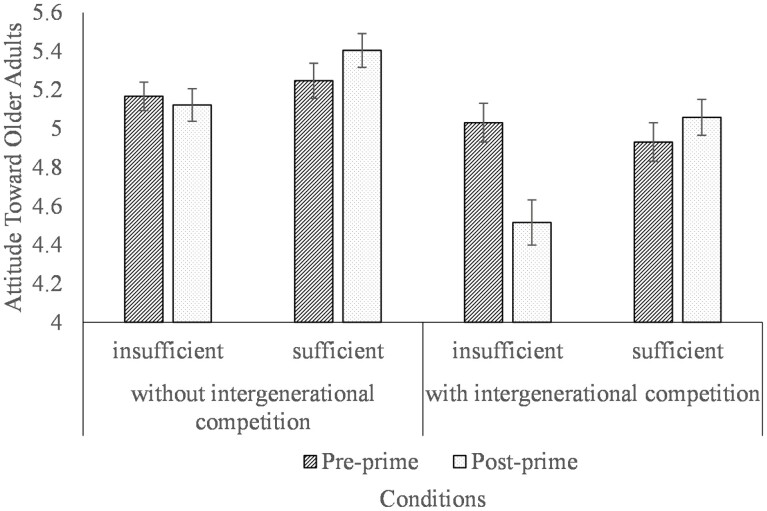
Attitudes towards older adults as a function of resource scarcity and perceived generational competition in Study 2. Error bars represent the standard error of the means.

A 2 (time: pre/postprime, within-subject) × 2 (levels of sufficiency) × 2 (salience of competition) mixed-model ANOVA on attitude scores (please refer to the supplementary for more detailed analysis) showed a significant three-way interaction effect (*F*_(1, 271)_ = 4.41, *p* = .037, partial η^2^ = 0.016. Simple-simple effect analysis revealed a significant decrease in attitudes from Wave 1 (*M* = 5.03, *SD* = 0.82) to Wave 2 (*M* = 4.51, *SD *= 0.96), only on the condition that both the insufficiency of pension and competition between generations were primed (*F*_(1, 271)_ = 23.69, *p* < .001, partial η^2^ = 0.08; Figure 2). Although participants’ attitudes did not change significantly between the two waves on the other combinations, such as only insufficiency of pension but without intergenerational competition (Wave 1: *M* = 5.17, *SD* = 0.62; Wave 2: *M* = 5.12, *SD* = 0.71; *F*_(1, 271)_ = 1.31, *p* = .253, partial η^2^ = 0.005), or only the intergenerational competition was primed with sufficient pension (Wave 1: *M* = 4.93, *SD* = 0.78; Wave 2: *M* = 5.06, *SD* = 0.72; *F*_(1, 271)_ = 0.20, *p* = .659, partial η^2^ < 0.001), or pension sufficiency without intergenerational competition (Wave 1: *M* = 5.25, *SD* = 0.79; Wave 2: *M* = 5.40, *SD* = 0.76; *F*_(1, 271)_ = 2.46, *p* = .118, partial η^2^ = 0.009). These results suggested that the pension insufficiency manipulation indeed evoked negative attitudes toward older adults under the competition salience condition, such that priming the salience of pension crisis, especially resource competition, diminished younger participants’ attitudes toward older adults. The results support Hypothesis 2.

## Study 3: Reducing Pension Tension by Facing the Future Self

In Study 3, we explored a potential future-self intervention to offset the deleterious effect of resource tension (as reflected by the manipulation of insufficient pension with intergenerational competition) on attitudes toward older adults. Previous research has shown that future-self conceptions influence decision-making: Interacting with computer renderings of one’s future-self increases the tendency to accept later monetary rewards over immediate ones (Hershfield et al., 2011). In a similar vein, priming participants with their future selves might increase their willingness to support the pension system, from which they would also benefit in the long term.

### Method

Before data collection, G*power analysis recommended 206 participants for conducting a between-subjects ANOVA (we set the effect size equal to 0.25, α error probability equal to 0.05, and test power equal to 0.90). We recruited 242 Chinese younger adults ages 18–28 (51% female, *M*_age_ = 20.66, *SD*_age_ = 2.05) from local comprehensive universities via convenience sampling in the year 2016. Participants received course credit for their participation in the present study.

Participants were introduced that they would perform a mobile app evaluation task, taking a “selfie” and using the app to alter their facial image. Participants entered one of the three conditions. In the *future-self-awareness* condition, the Aging Booth app aged participants’ faces by adding wrinkles and graying hair. In the *control* condition, the Mix Booth app altered participants’ faces in a neutral manner (e.g., changing hair color). Given the future-self intervention may simultaneously indicate an unpleasant self-image (e.g., the young participants may perceive the wrinkles as negative symbols), we additionally created the *negative self-image* condition to account for such potential negative components. In the *negative self-image* condition, the Fat Booth app altered participants’ faces to appear larger, widening them, and adding double chins.

After using the assigned app, participants reported their emotions through the Positive and Negative Affect scale (Watson et al., 1988; positive affect α = 0.819, negative affect α = 0.923) and the Identity subscale of the SIC Ageism scale (e.g., “Older people typically shouldn’t go to places where younger people hang out”) on a 6-Likert scale (“1-strongly disagree”; “6-strongly agree”; North & Fiske 2013a, 2013b, *Cronbach’s* α = 0.830), as a manipulation check, to ascertain that different self-image manipulations indeed elicit differential reactions (for detailed manipulation check results, see Supplemental Materials). Participants then received the same pension manipulation as Study 2 (sufficient pension without intergenerational competition/insufficient pension with intergenerational competition) and completed the same measure of attitudes toward older adults. Participants also completed a similar two-item manipulation check regarding the perceived status of Chinese pensions.

### Results and Discussion

**Figure 3.**
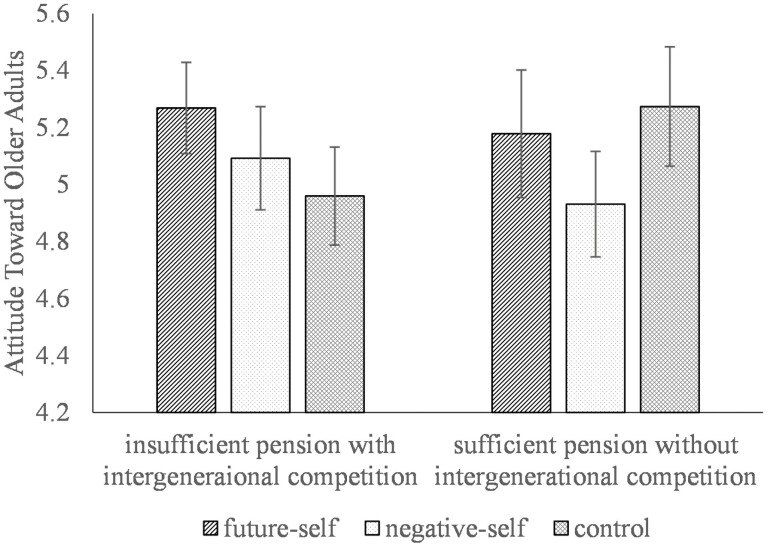
Attitudes towards older adults as a function of perceived pension scarcity and competition and interventions in Study 3. Error bars represent the standard error of the means.

Similar to Study 2, we collapsed perceptions of warmth and competence toward older adults into one overall attitude score (please refer to supplementary for details). A 3 (condition: future self/negative self/ control) × 2 (manipulation: sufficient pension without intergenerational competition and insufficient pension with intergenerational competition) ANOVA was conducted on the attitude score. The condition × manipulation two-way interaction was significant (*F*_(2, 236)_ = 3.57, *p* = .030, partial η^2^ = 0.029). Simple main effect analysis revealed that the effect of pension manipulation was significant only in the control condition (insufficient pension with intergenerational competition: *M* = 4.96, *SD* = 0.58; sufficient pension without intergenerational competition: *M* = 5.27, *SD* = 0.70; *F*_(1, 236)_ = 5.56, *p* = .019, partial η^2^ = 0.023) but not in the future-self-image condition (insufficient pension with intergenerational competition: *M* = 5.27, *SD* = 0.52; sufficient pension without intergenerational competition: *M* = 5.18, *SD* = 0.57; *F*_(1, 236)_ = 0.50, *p* = .478, partial *η*^2^ = 0.002) or the negative self-image condition (insufficient pension with intergenerational competition: *M* = 5.09, *SD* = 0.73; sufficient pension without intergenerational competition: *M* = 4.93, *SD* = 0.57; *F*_(1, 236)_ = 1.09, *p* = .297, partial η^2^ = 0.005). This result replicated findings from Study 2, which was similar to the control condition, such that priming with information on insufficient pension with intergenerational competition over pension heightened negative attitudes toward older adults (Figure 3).

For the future-self intervention, we investigated whether the future-self per se instead of its negative components (as reflected in the negative-self condition) could reduce negative ageist attitudes. Therefore, by comparing the attitudes toward older adults with the control condition, we tested and compared the effects of future-self and negative-self intervention, especially under the pension scarcity manipulation. First, we found marginally significant main effects of intervention under both sufficient (*F*_(2, 114)_ = 3.12, *p* = .048, partial η^2^ = 0.052) and insufficient (*F*_(2, 122)_ = 2.66, *p* = .07, partial η^2^ = 0.042) pension manipulations. More importantly, under the insufficient pension with intergenerational competition manipulation, post hoc analysis (with LSD criterion) revealed that only participants from the future-self-image condition (*M* = 5.27, *SD* = 0.52, *p* = .023), but not those who primed with a negative-self (*M* = 5.09, *SD* = 0.73, *p* = .327) expressed more positive attitudes toward older adults compared with participants from the control condition (*M* = 4.96, *SD* = 0.57).

Moreover, under the sufficient pension without intergenerational competition manipulation, post hoc analysis revealed that participants’ attitudes toward older adults in the future-self condition (*M* = 5.18, *SD* = 0.57, *p* = .490) were not significantly different from their counterparts in the control condition (*M* = 5.27, *SD* = 0.70). However, those who were primed with a negative self-image instead reported more negative attitudes towards older adults (*M* = 4.93, *SD* = 0.57, *p* = .016), compared with participants in the control condition. We further compared directly between the future-self and negative-self conditions. Results revealed that participants primed with their future-self expressed slightly more positive (but nonsignificant) attitudes toward older adults than their counterparts primed with their negative self under both insufficient (future-self: *M* = 5.27, *SD* = 0.52; negative-self: *M* = 5.09, *SD* = 0.73; *p* = .195), and sufficient manipulations (future-self: *M* = 5.18, *SD* = 0.57; negative-self: *M* = 4.93, *SD* = 0.57; *p* = .089).

Overall, Study 3 suggested that future-self priming among younger participants mollifies the deleterious effect of resource tension on attitudes toward older adults (see Figure 3). Moreover, this effect was achieved through a future-related self-perception rather than a negative self-perception. The result dovetails with studies suggesting that a future-self continuity intervention might activate a long-term mindset, strengthening the link between themselves and their future self (Hershfield et al., 2011). Under such circumstances, perhaps younger people grow more aware of one day benefitting themselves from the pension system, therefore increasing their short-term support for long-term gain, which supports Hypothesis 3.

## General Discussion

Comprising nearly 70,000 data points and both correlational survey and experimental methods, three studies identified an undesirable side effect of public pensions: exacerbating young adults’ resentment (via lowered warmth and competence perceptions) toward older generations. According to integrated threat theory (Croucher, 2017; Stephan & Stephan, 2000), this suggests a realistic, “dark side” of pensions, driven by competition for resources between younger and older adults. Although at first it may seem surprising that warmth and competence exhibited a similar pattern as one another in the current studies—given that prior work implied orthogonality between the two, and showed older people to be perceived as low in competence but high in warmth (e.g., Fiske et al., 2002)—warmth and competence dimensions have been shown to correlate highly when it comes to modern perceptions of older adults (North & Fiske, 2013b).

The current findings present at least three key considerations for future research on the globally aging population, age-based prejudice, and future-self thinking: (a) supporting a resource tension hypothesis of older adult attitudes; (b) implicating public pensions as a key resource underlying these tensions; and (c) identifying an effective future-self intervention to mitigate these tensions.

### Implicating Public Pensions as a Specific Resource Fostering Generational Tension

To our knowledge, the present paper is the first to identify public pension resources as a specific real-world mechanism explaining *why* population aging fosters ageism. As Intergroup Contact Theory predicts (i.e., more cross-generational opportunities should facilitate positive generational relations), studies find a positive association between the aging population and positive feelings toward older people over 70 across countries (Abrams et al., 2011). However, prior studies rarely considered pension expenditure or intergenerational resource competition in analyzing attitudes toward older adults.

Although other large-scale studies indeed find a negative association between nation-level older population and individual attitudes toward older adults (Löckenhoff et al., 2009; North & Fiske, 2015b; Zhang et al., 2016), these results mostly lack a more proximal predictor underscoring how this tension manifests “on the ground.” In other words, previous studies have offered a resource scarcity hypothesis to explain the association between population aging and ageism (i.e., greater aging population → greater intergenerational resource scarcity → increased negative attitudes toward older adults), but these studies do not measure directly the resource in question. Instead, they use population aging as a proxy for gauging generational resource tensions.

By contrast, the current paper focally implicates pensions as a specific resource tension driving ageism. Prior qualitative research suggests that pension expenditure performs as a proxy of intergenerational inequality (Hurley et al., 2017). Similarly, health care funds allocated to older adults incite ageist attitudes against them, even if these policies per se are well-intentioned (Ng et al., 2022). To date, most industrialized countries feature some form of social pension systems, funded by taxes on the working population. According to the competition-based ageism model, such redistribution of resources might yield increased concerns in the younger, working population who foot the bill for the pension system, thereby diminishing their attitudes toward their older adults. Thus, understanding the inadvertent tensions that might arise from these systems is a global concern.

### Resource Tensions Trump Cultural Values in Shaping Older Adult Perceptions

The current findings contribute to an ongoing scholarly subtopic exploring whether collectivistic cultures hold their older adults in higher esteem than Western ones. Some degree of evidence exists on both sides of this issue, including a recent paper showing that, on average, Eastern cultures do in fact hold more positive views of older adults than do Western ones using implicit (IAT) and certain explicit measures (Ackerman & Chopik, 2021), and a wide range of participant ages. Such evidence supports the aforementioned cultural values hypothesis—that Eastern collectivism and filial piety expectations generate elevated respect for societal older adults—and thus, reduced ageism (Nelson, 2009).

On the other hand, substantial evidence also exists for the resource-tension hypothesis, including large-scale studies utilizing different measures, contexts, and a host of different countries. Young adults in Eastern countries report no greater respect toward older adults (Löckenhoff et al., 2009). This likely derives from resource tensions, as argued in meta-analytic investigations (North & Fiske, 2015b). The current paper adds further support, showing that resource tensions affect generational relations equally in the East as prior work has shown within the West (North & Fiske, 2013b). In the modern world, generational resource scarcity is a heightened risk factor for ageism, regardless of the cultural values placed upon younger generations.

### Intervening Generational Tension via a Future-self Manipulation

Finally, the current results offer an effective intervention for generational tensions over pension resources: facing the future self. On the one hand, the current paper replicated the prior findings by Rittenour and Cohen (2016) that facing the future-self (with processed photos) may not change younger participants’ attitudes toward older adults when intergenerational tension was not salient. On the other hand, however, our work supports its feasibility under resource scarcity conditions, such that the future-self image can indeed enhance younger adults’ positive attitudes toward older adults. In other words, such future-self intervention might be more important when intergenerational tensions are emphasized. Compared with prevailing interventions on ageism, such as the PEACE program (Lytle & Levy, 2019; Lytle et al., 2021), our attempts at facing the future-self propose a simpler strategy that can be implemented by individuals themselves to modulate their ageist attitudes. Future work should continue elucidating the underlying mechanism of future-self thinking, such as age identification (Packer & Chasteen, 2006), aging anxiety (Rittenour & Cohen, 2016), or inclusion of outgroups in the self (Chen & Zhang, 2022)—all in the name of ameliorating generational relations, especially as the world population grows older and more multigenerational (North & Fiske, 2015b).

### Limitations

First, due to the changing topics involved in different waves of the ESS and the WVS data, the databases we used in Study 1 were derived in the years 2008 and 2014, which may not specifically identify the association between scarcity of pension and intergenerational tension at present and result in the smaller effect sizes and significant levels of the current analysis.

Moreover, a temporal consideration might affect the current findings. For example, participants recruited in Study 1 may be more impacted by unique social events (e.g., the economic crisis in 2008) compared with those from younger generations recruited in Studies 2 and 3 several years later. As a result, they might experience different intergenerational relationships and then form their ageist attitudes. Nevertheless, such results from Study 1 could be replicated at the micro-level with a more recent cohort (Studies 2 and 3), which would confirm the robustness of the pension-tension association across time frames. Considering the soaring aging population and changing world in the recent decade, the latest cross-country survey concerning intergenerational tension might provide more convincing evidence about the relationship between pension and tension.

In addition, participants were recruited from China (a typical collectivistic culture) in Study 2 and Study 3, so the generalizability of the present findings might be limited. Especially with growing opportunities for younger and middle-aged adults around the world to share their attitudes and values on the internet nowadays, the influence of cultural values per se may be hindered in our literature. More cross-cultural investigations might be needed.

Despite these limitations, our findings from World Value Survey and ESS (Study 1) implicate pension resources in underlying negative attitudes toward older adults cross-culturally. Study 2 further underscores generational resource tensions in modern ageism via experimental methods. Fortunately, a future-self intervention may be just what societies need to foster harmonious generational relations, pension-related, or otherwise (Study 3). In contemplating how to accommodate a globally aging and multigenerational population, policymakers, and scholars alike should consider tensions associated with intergenerational wealth transfer. Moreover, encouraging a rising younger generation to contemplate their own future suggested an adaptive path forward for solving this tricky generational puzzle, worldwide.

## Supplementary Material

igad080_suppl_Supplementary_Material

## Data Availability

Data sets, analytic methods, and experimental materials used are available from the corresponding author upon request. Studies reported in the manuscript were not pre-registered.
