# Supplementary material for: Pension Tension: Retirement Annuity Fosters Ageism Across Countries and Cultures
Source: Innov Aging. 2023 Jul 27;7(7):igad080. doi: 10.1093/geroni/igad080 (PMC10506169; doi:10.1093/geroni/igad080)
Supplement: igad080_suppl_Supplementary_Material [file igad080_suppl_supplementary_material.docx]

**Online Supplementary Material**

**Supplemental analyses of Study 2**

**Collapsing warmth and competence - ANOVA on the perception of older adults in Study 2**

A 2 (attitudes: warmth/competence, within-subject) × 2 (time: pre-/post-prime, within-subject) × 2 (levels of sufficiency) × 2 (salience of competition) mixed-model ANOVA on attitude scores showed that the 4-way interaction effect was not significant (F(1, 271) = .62, p = .43, partial η2 = .002). The 3-way interaction effect between dimensions, sufficiency, and salience of competition did not reach significance, either. However, a significant 3-way interaction effect between time, sufficiency, and salience of competition emerged, F(1, 271) = 4.41, p = .037, partial η2 = .016. Given that no main effect difference appeared between the two attitude dimensions, we used a composite attitude score of warmth and competence in the following analysis.

**Control Variables in Study 2**

We collected other demographic information and covariates, including age, gender, self-rated health, household income level, and personality (i.e., agreeableness and conscientiousness), while none of them influence our main effect.

**Supplemental analyses of Study 3**

**Manipulation Check in Study 3.**

One-way ANOVAs were conducted to test the effectiveness of self-image manipulations on positive affect negative affect and age identity respectively as manipulation checks. A significant main effect of self-image manipulation was found for positive affect (*F*_(2, 238)_ = 4.877, *p* =.008, partial *η*^2^ = .040), while negative affect (*F*_(2, 239)_ = 2.923, *p* =.056, partial *η*^2^ = .024) and age identity (*F*_(2, 239)_ = 2.620, *p* =.075, partial *η*^2^ = .021) was approaching significance (For descriptive results, please refer to the Table S3).

Post-hoc tests showed that participants from either future (*p* = .016) or negative (*p* = .038) self-images manipulations reported less positive affect than those who watched neutral self-images (control condition). Those who took negative self-images manipulation were more likely to experience negative feelings (*p* = .038, one-tailed) than their counterparts in the control condition. In addition, participants who took future self-images manipulation showed fewer ageist attitudes in the identity dimension (*p* = .036, one-tailed), which indicated they perceived older adults who behaved young as more tolerable, than those who watched negative self-images. The results suggested that both the future and negative self-images manipulations successfully aroused negative (or less positive) affect, and the future self-images manipulations further led to less identity-related ageism, indicating a successful manipulation.

**Collapsing warmth and competence - ANOVA on the perception of older adults in Study 3**

Similar to Study 2, a 3 (condition: future self/ negative self/ control) × 2 (manipulation: sufficient pension without intergenerational competition/ insufficient pension with intergenerational competition) × 2 (attitudes: warmth/ competence) mixed-design ANOVA on attitudes toward older adults revealed a nonsignificant 3-way interaction effect (F(2, 236) = .068, p =.934, partial η2 < .001). However, the 2-way interaction between condition and manipulation was significant (F(2, 236) = 3.57, p = .030, partial η2 = .029). Thus, we collapsed the perceptions of warmth and competence into one overall score in the following analysis.

**Table S1.** Description of WVS data (Study 1)

| **Country** | ***N*** |  | **Age** | **Education** | **Income** | **Female (%)** | **Pension (%)** | **Older Adults (%)** | **Gini index** | **GDP per capita ($1000)** | **Individualism** | **Warmth** | **Competence** | **Attitude** |
| --- | --- | --- | --- | --- | --- | --- | --- | --- | --- | --- | --- | --- | --- | --- |
| Australia | *978* | M | 50.47 | 6.76 | 5.19 | 55.3 | 3.50 | 20 | 35 | 51.83 | 90 | 2.96 | 2.22 | 2.59 |
|  |  | *SD* | *16.11* | *2.27* | *2.06* |  |  |  |  |  |  | *0.90* | *1.00* | *0.82* |
| Chile | *855* | M | 44.21 | 5.46 | 4.78 | 50.8 | 5.50 | 13 | 52 | 12.68 | 23 | 2.73 | 2.08 | 2.40 |
|  |  | *SD* | *16.32* | *2.05* | *1.68* |  |  |  |  |  |  | *0.94* | *1.12* | *0.82* |
| China | *1664* | M | 43.48 | 5.36 | 4.43 | 50.7 | 2.50 | 12 | 42 | 4.43 | 20 | 3.18 | 2.51 | 2.84 |
|  |  | *SD* | *14.84* | *2.34* | *1.88* |  |  |  |  |  |  | *0.79* | *1.04* | *0.73* |
| Ecuador | *1197* | M | 39.79 | 5.25 | 5.00 | 51.5 | 1.81 | 9 | 49 | 4.50 | 8 | 2.98 | 2.64 | 2.81 |
|  |  | *SD* | *16.13* | *2.16* | *1.88* |  |  |  |  |  |  | *1.13* | *0.99* | *0.87* |
| Estonia | *1458* | M | 48.40 | 6.62 | 4.36 | 55.6 | 9.00 | 23 | 36 | 14.30 | 60 | 2.46 | 2.21 | 2.33 |
|  |  | *SD* | *18.42* | *1.72* | *1.82* |  |  |  |  |  |  | *0.97* | *1.00* | *0.86* |
| Georgia | *1892* | M | 49.40 | 5.22 | 4.83 | 49.7 | 10.60 | 26 | 28 | 40.41 | 67 | 2.81 | 2.59 | 2.70 |
|  |  | *SD* | *17.59* | *2.25* | *1.80* |  |  |  |  |  |  | *0.97* | *1.02* | *0.86* |
| Ghana | *1540* | M | 30.94 | 4.34 | 4.85 | 49.7 | 1.30 | 6 | 43 | 1.33 | 20 | 2.71 | 2.58 | 2.64 |
|  |  | *SD* | *12.73* | *2.20* | *2.06* |  |  |  |  |  |  | *1.20* | *1.25* | *1.00* |
| Iraq | *1165* | M | 36.63 | 4.91 | 5.35 | 47.2 | 3.90 | 5 | 31 | 4.61 | 38 | 2.49 | 2.45 | 2.47 |
|  |  | *SD* | *13.38* | *2.62* | *1.86* |  |  |  |  |  |  | *1.08* | *1.14* | *0.97* |
| Japan | *1580* | M | 51.26 | 6.80 | 4.10 | 47.6 | 10.10 | 30 | 25 | 43.12 | 46 | 2.28 | 1.93 | 2.11 |
|  |  | *SD* | *15.38* | *1.76* | *2.76* |  |  |  |  |  |  | *0.90* | *0.87* | *0.78* |
| Jordan | *1188* | M | 39.83 | 5.20 | 5.00 | 49.8 | 2.00 | 6 | 35 | 4.37 | 30 | 2.87 | 2.77 | 2.82 |
|  |  | *SD* | *15.47* | *2.49* | *2.07* |  |  |  |  |  |  | *1.01* | *1.03* | *0.92* |
| Kuwait | *1060* | M | 36.43 | 6.78 | 5.96 | 33.9 | 2.70 | 4 | 30 | 40.09 | 25 | 2.90 | 2.88 | 2.89 |
|  |  | *SD* | *11.93* | *1.97* | *2.03* |  |  |  |  |  |  | *1.12* | *1.12* | *0.99* |
| Kyrgyzstan | *1192* | M | 39.12 | 6.76 | 5.47 | 51.3 | 6.60 | 7 | 33 | 0.88 | 38 | 2.40 | 2.45 | 2.42 |
|  |  | *SD* | *14.40* | *1.94* | *1.94* |  |  |  |  |  |  | *1.15* | *1.09* | *1.00* |
| Malaysia | *1219* | M | 40.07 | 5.05 | 6.00 | 48.8 | 3.75 | 8 | 46 | 8.75 | 26 | 2.86 | 2.61 | 2.73 |
|  |  | *SD* | *13.97* | *1.91* | *1.86* |  |  |  |  |  |  | *0.84* | *0.85* | *0.71* |
| Mexico | *1920* | M | 37.25 | 5.24 | 3.32 | 50.0 | 2.40 | 9 | 47 | 8.92 | 30 | 3.06 | 2.59 | 2.82 |
|  |  | *SD* | *14.96* | *2.32* | *2.44* |  |  |  |  |  |  | *1.19* | *1.29* | *1.04* |
| Dutch | *1461* | M | 54.48 | 5.95 | 4.64 | 51.3 | 5.00 | 22 | 31 | 46.77 | 80 | 3.00 | 2.24 | 2.62 |
|  |  | *SD* | *15.78* | *2.11* | *2.29* |  |  |  |  |  |  | *0.78* | *0.97* | *0.72* |
| New Zealand | *660* | M | 50.00 | 7.56 | 5.86 | 57.6 | 4.70 | 18 | 36 | 32.85 | 79 | 3.05 | 2.10 | 2.58 |
|  |  | *SD* | *16.43* | *1.37* | *2.79* |  |  |  |  |  |  | *0.93* | *1.21* | *0.87* |
| Nigeria | *1697* | M | 31.04 | 5.21 | 5.19 | 49.0 | 0.91 | 5 | 49 | 2.29 | 20 | 2.44 | 2.52 | 2.48 |
|  |  | *SD* | *11.57* | *2.18* | *2.12* |  |  |  |  |  |  | *1.17* | *1.08* | *0.92* |
| Pakistan | *1164* | M | 34.48 | 4.02 | 5.51 | 47.9 | 0.50 | 6 | 30 | 1.02 | 14 | 2.78 | 3.13 | 2.95 |
|  |  | *SD* | *11.89* | *2.25* | *2.15* |  |  |  |  |  |  | *1.35* | *1.10* | *1.02* |
| Peru | *1137* | M | 39.04 | 5.69 | 4.69 | 49.0 | 1.93 | 9 | 48 | 5.08 | 16 | 2.90 | 2.22 | 2.56 |
|  |  | *SD* | *16.20* | *2.16* | *1.81* |  |  |  |  |  |  | *1.06* | *1.21* | *0.94* |
| Philippines | *1183* | M | 42.65 | 5.61 | 4.18 | 50.4 | 1.50 | 7 | 43 | 2.14 | 32 | 2.67 | 2.45 | 2.56 |
|  |  | *SD* | *15.56* | *2.44* | *2.46* |  |  |  |  |  |  | *1.20* | *1.24* | *0.98* |
| Poland | *858* | M | 47.56 | 5.62 | 4.48 | 54.4 | 11.90 | 19 | 33 | 12.30 | 60 | 2.60 | 2.18 | 2.39 |
|  |  | *SD* | *17.36* | *2.12* | *1.88* |  |  |  |  |  |  | *1.10* | *1.11* | *0.96* |
| Romania | *1368* | M | 47.93 | 6.35 | 4.84 | 56.1 | 9.50 | 20 | 27 | 8.14 | 30 | 2.88 | 2.33 | 2.61 |
|  |  | *SD* | *17.11* | *1.87* | *2.13* |  |  |  |  |  |  | *1.14* | *1.21* | *0.97* |
| Russia | *2154* | M | 45.22 | 6.54 | 4.28 | 54.7 | 6.90 | 18 | 40 | 10.71 | 39 | 2.56 | 2.41 | 2.49 |
|  |  | *SD* | *17.18* | *1.80* | *1.77* |  |  |  |  |  |  | *1.13* | *1.13* | *0.98* |
| Slovenia | *979* | M | 49.17 | 5.70 | 4.93 | 58.0 | 11.20 | 22 | 31 | 22.90 | 27 | 2.69 | 2.03 | 2.36 |
|  |  | *SD* | *17.54* | *2.13* | *1.77* |  |  |  |  |  |  | *0.92* | *0.94* | *0.79* |
| Spain | *1009* | M | 46.30 | 4.72 | 4.52 | 50.3 | 8.20 | 22 | 35 | 29.73 | 51 | 2.62 | 2.05 | 2.34 |
|  |  | *SD* | *17.73* | *2.17* | *1.61* |  |  |  |  |  |  | *1.09* | *1.20* | *0.98* |
| Sweden | *1102* | M | 47.81 | 7.09 | 5.41 | 52.0 | 8.20 | 25 | 25 | 49.38 | 71 | 2.74 | 2.64 | 2.69 |
|  |  | *SD* | *19.14* | *1.92* | *1.83* |  |  |  |  |  |  | *0.96* | *0.95* | *0.78* |
| Egypt | *1500* | M | 40.60 | 4.20 | 4.27 | 67.8 | 3.00 | 7 | 31 | 2.80 | 38 | 2.93 | 2.88 | 2.91 |
|  |  | *SD* | *15.29* | *2.84* | *2.01* |  |  |  |  |  |  | *1.00* | *1.13* | *0.96* |
| United States | *2134* | M | 49.10 | 7.78 | 5.17 | 51.7 | 6.80 | 18 | 41 | 48.36 | 91 | 2.85 | 2.19 | 2.52 |
|  |  | *SD* | *16.79* | *1.29* | *1.91* |  |  |  |  |  |  | *0.92* | *1.00* | *0.82* |
| Uruguay | *914* | M | 45.16 | 4.61 | 4.53 | 52.6 | 8.79 | 18 | 45 | 11.53 | 36 | 2.87 | 2.17 | 2.52 |
|  |  | *SD* | *18.21* | *2.02* | *1.79* |  |  |  |  |  |  | *1.08* | *1.19* | *0.92* |

**Table S2**. Description of ESS data (Study 1)

| **Country** | ***N*** |  | **Age** | **Education** | **Household Income** | **Female (%)** | **Pension (%)** | **Older Adults (%)** | **Gini index** | **GDP per capita ($)** | **Warmth** | **Competence** | **Attitude** |
| --- | --- | --- | --- | --- | --- | --- | --- | --- | --- | --- | --- | --- | --- |
| Belgium | *1556* | M | 47.04 | 3.22 | 7.45 | 49.9 | 11.1 | 18.87 | 28.4 | 48106.89 | 2.85 | 2.51 | 2.68 |
|  |  | *SD* | *18.20* | *1.38* | *2.37* |  |  |  |  |  | *0.81* | *0.87* | *0.72* |
| Czech | *1427* | M | 47.91 | 3.06 | 3.43 | 52.1 | 7.8 | 14.74 | 26.3 | 22804.58 | 2.74 | 2.06 | 2.40 |
|  |  | *SD* | *17.15* | *0.73* | *1.55* |  |  |  |  |  | *0.99* | *1.01* | *0.83* |
| Germany | *2260* | M | 49.59 | 3.52 | 4.59 | 46.9 | 12.1 | 20.02 | 31.1 | 45427.15 | 2.73 | 2.30 | 2.52 |
|  |  | *SD* | *16.90* | *1.07* | *2.52* |  |  |  |  |  | *0.81* | *0.97* | *0.74* |
| Denmark | *1355* | M | 49.68 | 3.66 | 6.00 | 47.2 | 11.7 | 15.96 | 25.2 | 64322.06 | 3.15 | 2.52 | 2.83 |
|  |  | *SD* | *16.78* | *1.21* | *2.77* |  |  |  |  |  | *0.76* | *0.85* | *0.67* |
| Estonia | *1364* | M | 48.59 | 6.02 | 3.98 | 58.2 | 17.3 | 31.90 | 31.9 | 18227.12 | 2.81 | 6.90 | 2.85 |
|  |  | *SD* | *18.76* | *1.27* | *2.71* |  |  |  |  |  | *0.84* | *0.85* | *0.73* |
| Spain | *1542* | M | 47.64 | 2.46 | 4.87 | 52.9 | 9.3 | 16.81 | 34.2 | 35366.26 | 3.09 | 2.57 | 2.83 |
|  |  | *SD* | *18.35* | *1.46* | *2.49* |  |  |  |  |  | *0.89* | *0.99* | *0.78* |
| Finland | *1987* | M | 48.29 | 3.22 | 5.94 | 50.9 | 10.3 | 16.55 | 27.8 | 53544.04 | 3.02 | 2.71 | 2.86 |
|  |  | *SD* | *17.81* | *1.41* | *2.74* |  |  |  |  |  | *0.69* | *0.77* | *0.60* |
| France | *1816* | M | 48.56 | 3.13 | 5.83 | 54.1 | 13.3 | 16.55 | 33.0 | 45334.11 | 2.83 | 2.46 | 2.65 |
|  |  | *SD* | *18.06* | *1.46* | *2.84* |  |  |  |  |  | *0.83* | *0.92* | *0.74* |
| UK | *1974* | M | 48.81 | 3.25 | 5.34 | 53.3 | 10.1 | 16.25 | 34.1 | 47287.00 | 2.97 | 2.34 | 2.65 |
|  |  | *SD* | *17.71* | *1.71* | *3.00* |  |  |  |  |  | *0.80* | *0.88* | *0.72* |
| Greece | *1220* | M | 45.47 | 2.94 | 5.67 | 54.1 | 13.1 | 18.64 | 33.6 | 31997.28 | 3.04 | 1.95 | 2.49 |
|  |  | *SD* | *16.49* | *1.45* | *2.35* |  |  |  |  |  | *0.91* | *1.09* | *0.82* |
| Croatia | *1049* | M | 48.11 | 3.13 | 4.97 | 54.3 | 9.3 | 17.41 | 32.6 | 16296.81 | 2.80 | 1.96 | 2.38 |
|  |  | *SD* | *18.24* | *1.14* | *2.88* |  |  |  |  |  | *1.11* | *1.25* | *1.03* |
| Hungary | *1101* | M | 48.60 | 3.00 | 5.25 | 55.4 | 10.7 | 15.95 | 27.5 | 15753.47 | 3.09 | 3.02 | 3.05 |
|  |  | *SD* | *19.00* | *1.12* | *2.43* |  |  |  |  |  | *0.92* | *0.93* | *0.79* |
| Ireland | *1536* | M | 47.96 | 3.35 | 4.60 | 54.0 | 6.4 | 10.74 | 30.9 | 61262.10 | 3.33 | 2.68 | 3.01 |
|  |  | *SD* | *17.64* | *1.57* | *2.51* |  |  |  |  |  | *0.71* | *0.87* | *0.65* |
| Latvia | *1574* | M | 49.92 | 3.23 | 4.61 | 63.2 | 5.7 | 17.78 | 37.2 | 16422.11 | 2.78 | 2.82 | 2.80 |
|  |  | *SD* | *18.24* | *1.13* | *2.44* |  |  |  |  |  | *0.91* | *0.95* | *0.80* |
| Netherlands | *1540* | M | 49.35 | 3.15 | 6.09 | 54.0 | 11.2 | 14.77 | 29.3 | 57644.48 | 2.90 | 2.54 | 2.72 |
|  |  | *SD* | *17.06* | *1.34* | *2.72* |  |  |  |  |  | *0.78* | *0.86* | *0.69* |
| Poland | *1244* | M | 45.49 | 3.17 | 6.19 | 53.5 | 11.5 | 13.23 | 33.5 | 13996.03 | 2.98 | 1.87 | 2.43 |
|  |  | *SD* | *18.37* | *1.02* | *2.78* |  |  |  |  |  | *0.82* | *0.98* | *0.72* |
| Portugal | *974* | M | 54.01 | 1.85 | 4.36 | 61.2 | 12.7 | 17.98 | 36.6 | 24847.55 | 2.84 | 2.64 | 2.74 |
|  |  | *SD* | *19.10* | *1.28* | *1.90* |  |  |  |  |  | *0.99* | *1.10* | *0.93* |
| Romania | *1588* | M | 47.14 | 2.86 | 5.48 | 53.8 | 7.3 | 15.53 | 36.4 | 10435.04 | 2.58 | 2.57 | 2.57 |
|  |  | *SD* | *17.32* | *1.11* | *3.29* |  |  |  |  |  | *1.10* | *1.05* | *0.95* |
| Sweden | *1684* | M | 48.04 | 3.20 | 6.99 | 49.5 | 11.3 | 17.75 | 28.1 | 56152.55 | 3.12 | 2.54 | 2.83 |
|  |  | *SD* | *18.54* | *1.29* | *2.46* |  |  |  |  |  | *0.69* | *0.84* | *0.61* |
| Slovenia | *1006* | M | 47.22 | 3.16 | 5.07 | 54.8 | 9.5 | 16.26 | 23.7 | 27483.34 | 2.98 | 2.06 | 2.52 |
|  |  | *SD* | *18.27* | *1.09* | *2.89* |  |  |  |  |  | *0.87* | *1.00* | *0.78* |

**Table S3**. Results of Self-Images Manipulation in Study 3

| **Self-Images Manipulations** | **Positive Affect** | | **Negative Affect** | | **Ageism of Identity** | |
| --- | --- | --- | --- | --- | --- | --- |
|  | *M* | *SD* | *M* | *SD* | *M* | *SD* |
| Future | 2.17 | 0.58 | 1.96 | 0.80 | 2.09 | 0.77 |
| Negative | 2.20 | 0.69 | 2.02 | 0.92 | 2.38 | 1.01 |
| Neutral | 2.47 | 0.78 | 1.72 | 0.88 | 2.20 | 0.65 |
